# A data-driven approach to decode metabolic dysfunction-associated steatotic liver disease

**DOI:** 10.1016/j.aohep.2023.101278

**Published:** 2024

**Authors:** Maria Jimenez Ramos, Timothy J. Kendall, Ignat Drozdov, Jonathan A. Fallowfield

**Affiliations:** a Centre for Inflammation Research, Institute for Regeneration and Repair, University of Edinburgh, Edinburgh BioQuarter, 4-5 Little France Drive, Edinburgh EH16 4UU, UK; b Edinburgh Pathology, University of Edinburgh, 51 Little France Crescent, Old Dalkeith Rd, Edinburgh EH16 4SA, UK; c Bering Limited, 54 Portland Place, London, W1B 1DY, UK

**Keywords:** NAFLD, MASLD, Big data, Artificial intelligence, Machine Learning, Precision medicine

## Abstract

Metabolic dysfunction-associated steatotic liver disease (MASLD), defined by the presence of liver steatosis together with at least one out of five cardiometabolic factors, is the most common cause of chronic liver disease worldwide, affecting around one in three people. Yet the clinical presentation of MASLD and the risk of progression to cirrhosis and adverse clinical outcomes is highly variable. It, therefore, represents both a global public health threat and a precision medicine challenge. Artificial intelligence (AI) is being investigated in MASLD to develop reproducible, quantitative, and automated methods to enhance patient stratification and to discover new biomarkers and therapeutic targets in MASLD. This review details the different applications of AI and machine learning algorithms in MASLD, particularly in analyzing electronic health record, digital pathology, and imaging data. Additionally, it also describes how specific MASLD consortia are leveraging multimodal data sources to spark research breakthroughs in the field. Using a new national-level ‘data commons’ (SteatoSITE) as an exemplar, the opportunities, as well as the technical challenges of large-scale databases in MASLD research, are highlighted.

## 1 Introduction

Metabolic dysfunction-associated steatotic liver disease (MASLD), previously termed non-alcoholic fatty liver disease (NAFLD), is characterized by the presence of liver steatosis and at least one of the five cardiometabolic criteria proposed in a multi-society Delphi consensus statement [1]. Importantly, other causes of steatosis, including increased alcohol intake, must be absent. Metabolic dysfunction-associated steatohepatitis (MASH), previously termed non-alcoholic steatohepatitis (NASH), is the progressive stage of the disease distinguished by the presence of lobular inflammation, hepatocyte ballooning, and an increased risk of liver fibrosis. In some instances, fibrosis progression can lead to cirrhosis and the development of hepatocellular carcinoma (HCC). The presence of certain genetic variants, such as single nucleotide polymorphisms in patatin-like phospholipase domain-containing protein 3 (PNPLA3), hydroxysteroid 17β dehydrogenase 13 (HSD17B13), or transmembrane 6 superfamily member 2 (TM6SF2) genes has also been associated with an increased risk of MASLD development, progression, and unfavorable prognosis [2], [3], [4]. Currently, MASLD represents the main cause of chronic liver disease and the leading indication for liver transplantation, affecting ∼30% of the global population [5]. Epidemiological modeling predicts a substantial increase in prevalence, clinical burden, and socioeconomic costs in the coming years – a public health threat that no country appears well prepared to address [6].

Crucially, the severity of fibrosis in MASLD is strongly associated with an increased risk of overall and disease-specific morbidity and mortality [7]. The most common cause of death in people with MASLD is cardiovascular disease, followed by extra-hepatic malignancy, then liver-related mortality [8,9]. These findings reflect the range of comorbidities in MASLD and highlight the need for a multidisciplinary approach to the disease [1].

Despite substantial advances in our understanding of disease pathogenesis, there are still no approved therapies for MASLD, and many drugs have shown limited efficacy in clinical trials, especially in patients with cirrhosis [10]. Given the complexity of disease pathogenesis, combination drug therapy may be required to improve patient outcomes [11], but the optimal combinations or treatment regimens are unknown. Additionally, there is an unmet need for non-invasive biomarkers to accurately diagnose, stage, and monitor the progression of MASLD and reduce or obviate the necessity for a liver biopsy in clinical practice and pharmacological studies. Moreover, the heterogeneity in progression and prognosis of MASLD calls for novel approaches to disease stratification and prediction of individual risk of clinical outcomes; this may require a re-evaluation of MASLD, viewed through the prism of the new nomenclature, with integration of multimodal information including demographic, pathological, genetic/multi-omic, environmental, and electronic health record (EHR) data to understand patient trajectories and define discrete subphenotypes to enable precision medicine in MASLD [12].

In this review, we discuss how large-scale patient data and emerging artificial intelligence (AI) approaches are increasingly being leveraged in the MASLD field, in the quest for new diagnostic biomarkers, efficacious drug targets, and improved patient stratification and prognostication methods. A national-level multimodal database – SteatoSITE [13] – is used as an exemplar to demonstrate the utility and scope of an integrated data-driven approach, to highlight the technical challenges, and to illustrate possible future directions.

## 2 Big data classes and their utility in MASLD research

**Fig. 1:**
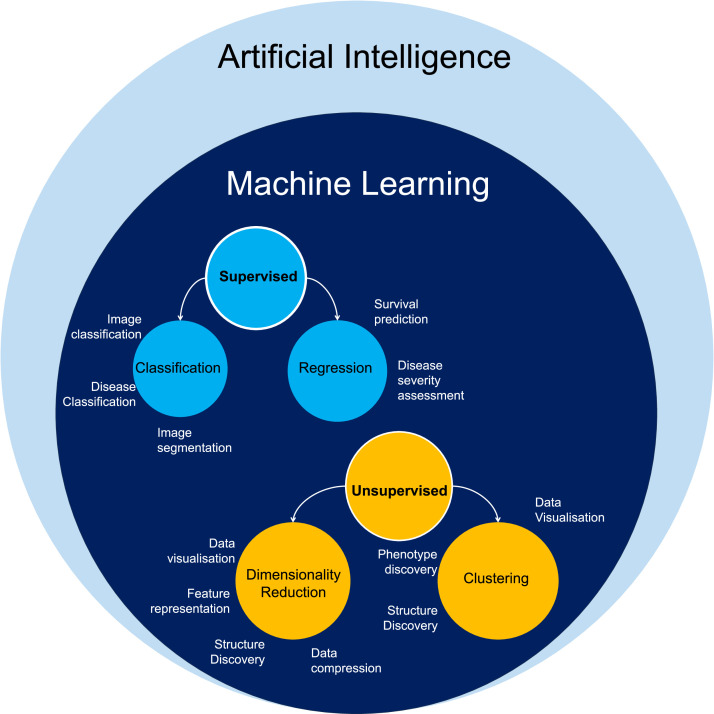
Schematic representation of the relationship between Artificial Intelligence and Machine Learning (ML), with ML algorithm categories and their applications.

AI is a large and rapidly growing field, using computer software that mimics human cognitive abilities to perform complex tasks. Machine Learning (ML) is an application of AI that enables computers to learn and recognize patterns from data to make decisions and predictions (Fig. 1). The two broad categories of ML algorithms are: supervised (the computer learns from both input data and corresponding correct answers) and unsupervised (the computer only processes input data). Their main advantage is that they can recognize unique data patterns and include multiple components to create new disease classifications and predictive models through linkage to outcomes [14]. AI/ML applications in liver disease research has increased in recent years, including in studies of MASLD to address the challenges of pathophysiological complexity and heterogeneity of presentation and patient outcomes.

### 2.1 Electronic health record data

Electronic Health Records (EHRs) are digital repositories of comprehensive patient health information, stored in standardized formats for efficient retrieval and sharing among healthcare providers. In both the United States (US) and the European Union, the adoption of EHRs has become nearly ubiquitous in both acute hospital and primary care settings [15]. EHR systems typically encompass administrative and healthcare utilization data, demographic details, diagnostic and procedural codes, laboratory results, pathology assessments, and prescribed medications.

The increased accessibility of EHRs for research has opened new avenues for large-scale observational studies and the application of AI/ML in MASLD, especially for predicting the risk of MASLD development or refining its diagnosis [16], [17], [18], [19], [20], [21]. For example, Fialoke *et al*. used one of the largest US-based EHR resources (from Optum Analytics), which integrates healthcare data from 50 provider organizations treating more than 80 million patients, for a supervised ML classification of MASLD patients to predict the health status of the patient cohort. The inclusion of time-stamped data also facilitates longitudinal profiling of candidate biomarkers and the identification of potential predictor variables associated with clinical outcomes. Typically, EHR data is characterized by noisy, sparse, and irregularly timed observations, which poses a challenge for phenotype discovery in clinical data, although computational ingenuity can overcome this [22], [23], [24]. To date, there are very few AI/ML-based studies in MASLD that have leveraged temporally defined EHR data to gain insights into disease progression or prognosis. Vandrome *et al*. [25] used data mining techniques to search for MASLD subtypes in a hospital database cohort of 13,290 patients, identified using electronic signatures of the disease. Using hierarchical clustering, they identified five distinct subtypes of patients. Notably, two of the major groups exhibited fewer comorbidities and favorable outcomes, whereas a minority within the three smaller subtypes displayed more severe comorbidities and poorer outcomes.

### 2.2 ‘Omics data

While EHR-based studies involve a substantial number of patients, none have integrated 'omics data to identify potential disease signatures for patient prediction and stratification. Despite this gap, several smaller studies have made efforts to address the issue.

Utilizing datasets from the Gene Expression Omnibus (GEO) [26], some researchers have conducted differential gene expression analyses, followed by network analysis and the application of ML algorithms. This methodology has enabled the identification of parsimonious gene signatures with a good Area Under Receiver Operating Characteristic Curve (AUROC) for the diagnosis of MASLD [27,28]. Sen *et al*. [29] employed transcriptomics of whole liver tissue and serum metabolomics from a cohort based on genome-scale metabolic models to identify dysregulated glycosphingolipid pathways across the disease spectrum. In the study by Luo *et al*. [30] the focus was on identifying serum biomarkers associated with liver fibrosis in patients with MASH. Although they identified key proteins linked to fibrosis and liver injury, they were unable to establish a protein panel capable of distinguishing between early and late fibrosis.

The investigation of interactions between MASH and other diseases has yielded notable findings. Qian *et al*. [31] defined a 20-gene signature predicting fibrosis progression in MASLD and HCV patients over five years, validated with an AUROC of 0.86. They also identified potential antifibrotic drug candidates and BCL2 as a therapeutic target. Additionally, Fujiwara *et al*. [32] developed a 133-gene signature for MASLD patients developing HCC, validated in a separate HCC cohort, and converted into a four-parameter blood-based panel (comprising XCL1, GRN, ANGPT2, and MET).

More advanced models have also been explored. Conway *et al*. [33] utilized ML on clinical trial data (STELLAR 3 and 4) to establish a prognostic five-gene signature predicting progression to cirrhosis and liver-related events in MASH patients, correlating with histological features. Deep learning (DL) was also investigated, outperforming other algorithms with an AUROC >0.80 in identifying genes associated with MASL to MASH progression [34]. Among the final 39 candidates identified, 11 were linked to HCC and survival rate.

### 2.3 Imaging data

Non-invasive imaging techniques have been employed in MASLD research and clinical settings. Advanced magnetic resonance imaging (MRI), including proton-density fat fraction (PDFF) and MR elastography (MRE), facilitates accurate quantification of steatosis and fibrosis for MASH assessment [35]. Recent applications of supervised ML and DL in medical imaging enhance automation, enabling more precise diagnosis. Training these models can unveil abnormal patterns beyond human perception, enhancing the efficiency of non-invasive diagnostic procedures. Studies have utilized ML to predict MRE liver stiffness, achieving an AUROC of 0.84 when combined with clinical data [36]. In a study by Schawkat *et al*. [37] MRI was employed to explore the viability of assessing liver scarring by integrating texture analysis, a method for extracting information from grey-level intensity within an image, with a supervised ML algorithm. Their results demonstrated a classification accuracy of 87.7%, equivalent to the performance level of MRE.

AI has also been utilized in the analysis of computerized tomography (CT) scans, which can measure liver fat content. Currently, there are no standardized approaches for manually delineating the region of interest (ROI), although some proposals exist, as outlined in Starekova *et al*. [38]. AI can facilitate automated liver segmentation, contributing to standardized CT analysis methods for MASLD patients. Several studies have already achieved this, demonstrating a robust and significant correlation [39], [40], [41]. Notably, a semi-automated DL-augmented method has been used on MRI-acquired 3D liver images to facilitate modeling of resectional surgery for liver cancer [42].

Liver ultrasound scans are a standard non-invasive diagnostic tool for chronic liver diseases, including MASLD, but are influenced by examiner subjectivity and exhibit reduced sensitivity when the liver contains less than 20–30% fat [43]. Limited studies on AI's application for predicting and classifying MASLD patients indicate promising results with excellent AUROC scores [44], [45], [46]. Additionally, ML algorithms integrated with transient elastography (TE) have been employed to predict liver fibrosis and MASLD in large clinical trial/cohort studies [47], [48], [49].

### 2.4 Digital pathology data

Despite these promising results, the gold standard for diagnosis of MASLD and MASH requires a liver biopsy where steatosis, inflammation, hepatocyte ballooning, and fibrosis are assessed. Whilst a clinical histopathological diagnosis is made by a pathologist integrating all histological features, in a research setting there are two main systems for ordinal scoring of the cardinal histological features. Features of disease activity can be evaluated with the NAFLD Activity Score (NAS) and the stage scored using the NASH Clinical Research Network (CRN) system [50], or disease activity assessed using the SAF (steatosis, activity, and fibrosis) system that scores ballooning and inflammation using different criteria but incorporates the same NASH-CRN stage. The architects of the NAS system explicitly state that a NAS score should not be used to define a diagnosis of steatohepatitis, although a NAS≥4 is often erroneously used for such a purpose. A system based upon score assignment by an observer is inherently subjective with inter- and intra-observer variation. To make the assignment of disease activity or stage scores more reproducible, AI methodologies are being developed to automate feature scoring.

HistoIndex (https://www.histoindex.com/) uses second harmonic generation (SHG) and two-photon excitation (TPE) microscopy with AI analysis to undertake histological assessment of unstained tissue sections [51]. Computationally derived qFIBS scores [52], that are analogous to the pathologist-assigned NAS components and NASH-CRN stage, can be generated, and this tool was used in an international multicentre study to assess lobular inflammation, steatosis, fibrosis, and hepatocyte ballooning. qFIBS had a strong correlation with each component of NAS (*P* < 0.001) and had an AUROC between 0.82 and 0.986 for each component.

PathAI (https://www.pathai.com) has developed a ML model that uses the digital images of biopsies for automated and quantitative assessment of a disease. The team used DL to predict NAS and fibrosis across three different clinical trials of advanced MASH [53]. Their findings revealed a significant correlation between the NAS scores and fibrosis and their ML model. Additionally, they also developed a new metric called Deep Learning Treatment Assessment (DELTA) Liver Fibrosis Score, designed to capture the change in fibrosis patterns from before to after the implementation of a treatment.

MorphoQuant^TM^ (https://biocellvia.com/) also uses standard stained sections from biopsies to quantify the collagen fibres, as well as the perivascular and septal percentage of collagen. It is AI-based and relies on morphometric recognition and no training is required. MorphoQuant^TM^ successfully quantified macrosteatosis, inflammation, and fibrosis in an automated manner in a mouse MASH model [54].

While the models described above used hematoxylin and eosin (H&E)-stained sections or unstained slides, PharmaNest (https://www.fibronest.com) developed the FibroNest AI algorithm, capable of analysing many different tinctorial stains to automatically quantify fibrosis and inflammation. Specifically, it can quantify collagen amount, structure, and the morphometric traits of their fibres, thereby providing a complete evaluation of fibrosis. They successfully predicted the development of HCC from MASLD through histopathology imaging studies [55]. Moreover, they also used their AI tool to assess fibrosis in a mouse study evaluating semaglutide [56]. Despite not observing a significant change in total fibrosis, their AI-based system revealed an amelioration of the collagen network architecture after treatment. While the total area of collagen remained unaltered, the treatment prevented its further accumulation.

In addition to tools to computationally replicate subjective ordinal feature scoring, methods have been developed to quantify features with continuous metrics. The earliest application of digital pathology in this area was the quantification of scarring in stained sections using simple colour thresholding [57], and AI-based classifiers have subsequently been developed to undertake the same task and provide a metric that complements the ordinal scar staging. Such classifiers are relatively easy to develop using open-source tools and have therefore been developed and used in a study-specific manner [13] that limits their generalizability and widespread application.

These studies show the importance of AI in enabling the stratification and automated quantification of key histopathological parameters in the diagnosis of MASLD. However, to maximize value it is important that such data is integrated with other diverse data sources, including EHRs, laboratory results, and genomic (and other ‘omics) profiles. There are several initiatives that are currently creating resources that store and analyze multimodal, multiscale information to elucidate new patient subphenotypes, identify new biomarkers and therapeutic targets.

## 3 Academia-industry research consortia in MASLD

AI-based approaches to understanding complex diseases are enabled by accessible large-scale multimodal datasets. The Foundation for the National Institute of Health (FNIH) initiative Non-invasive Biomarkers of Metabolic Liver Disease (NIMBLE) is a multi-stakeholder project to support regulatory approval of MASH-related biomarkers [58]. The diagnostic performance of five blood-based panels was evaluated in an observational cohort (*n* = 1073) covering the full spectrum of MASLD [59]. Multiple biomarkers met prespecified performance metrics. NIS4® had an AUROC of 0.81 for ‘at-risk’ MASH (steatohepatitis and fibrosis stage ≥F2). The AUROCs of the ELF^TM^ test, PROC3, and FibroMeter VCTE^TM^ for clinically significant fibrosis (≥F2), advanced fibrosis (≥F3), or cirrhosis (F4), respectively, were all ≥0.8.

The Liver Investigation: Testing Marker Utility in Steatohepatitis (LITMUS) consortium, supported by the European NAFLD registry [60], aims to develop, validate, and progress biomarkers for diagnosing, risk stratifying, and monitoring MASLD/MASH progression and fibrosis stage. The initiative involves a collaborative effort among end-users (clinicians and the pharmaceutical industry), independent academics specializing in medical test evaluation, and biomarker researchers and developers from academic or commercial backgrounds. Leveraging large-scale patient cohorts, bioresources and multi-omics datasets. The goal is to establish a definitive and impartial evaluation platform for these biomarkers. The LITMUS investigators developed prediction models using supervised ML techniques, that improved the detection of MASH and at-risk MASH [61]. They also created a proteo-transcriptomic map of MASLD signatures and generated a composite model comprising four proteins (ADAMTSL2, AKR1B10, CFHR4 and TREM2), body mass index and type 2 diabetes mellitus status to identify at-risk steatohepatitis [62]. LITMUS has recently added an imaging study where they will evaluate different MRI and elastography modalities against liver histology in MASLD [63].

TARGET-NASH, a longitudinal observational study, tracks patients under usual clinical care for MASLD/MASH in both academic and community settings [64]. The dataset is essential for establishing a baseline and assessing the impact of current practice guidelines, management, and new therapies on patients with various medical outcomes. The study's unique design, involving three years of retrospective analysis of MASH patients followed by at least five years of prospective enrolment, enables a comprehensive understanding of the disease's natural history. The TARGET-NASH cohort has allowed the validation of a clinical risk-based classification system [65], among other studies [66], [67], [68].

## 4 SteatoSITE

**Fig. 2:**
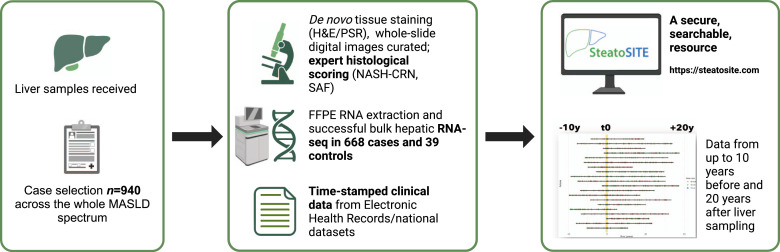
SteatoSITE Data Commons overview. The right panel includes a schematic diagram in which horizontal lines represent individual patient timelines decorated with a variable amount of multimodal data preceding or following the date of liver tissue sampling (time zero, indicated by the vertical yellow line). MASLD, metabolic dysfunction-associated steatotic liver disease; H&E, hematoxylin and eosin; PSR, picro-sirius red; NASH-CRN, Non-alcoholic steatohepatitis-Clinical Research Network; SAF, Steatosis, Activity, Fibrosis; FFPE, formalin-fixed paraffin-embedded; RNA-seq, RNA-sequencing.

The aforementioned consortia have compiled large multicentric prospective datasets. However, this presents potential disadvantages, including selection bias, loss to follow-up, and long duration to accumulate clinical outcomes. In contrast, SteatoSITE (https://www.steatosite.com) is a retrospective, multimodal MASLD database (Fig. 2) [13]. SteatoSITE includes curated whole-slide images of H&E and picro-sirius red-stained liver sections, accompanying histological assessments (NAS, SAF, NASH-CRN, collagen % area), bulk hepatic RNA-sequencing (RNA-seq), and rich EHR data from a cohort of *n* = 940 adult patients who had previously undergone either needle biopsy (*n* = 659), explant (*n* = 56) or liver resection (*n* = 225) between January 2000 and October 2019. Cases across the whole MASLD spectrum were identified from three of the four NHS Scotland Biorepositories (Lothian, Greater Glasgow & Clyde, and Grampian), representing 12 of the 14 territorial Health Boards. Covering a span of ten years before the tissue sampling date until May 2020, the dataset encompasses over 5.67 million days (∼15,547 years) of comprehensive routine clinical information derived from EHRs (including demographic data, International Classification of Diseases (ICD)-9/10 and OPCS Classification of Interventions and Procedures version 4 (OPCS-4) codes, laboratory results, and medication history).

SteatoSITE is a resource that can support multiple facets of MASLD research [13] and fulfils the FAIR attributes (Findability, Accessibility, Interoperability, and Reuse of digital assets) that underpin a ‘data commons’ [69]. One research avenue is the use of the extensive histopathological dataset linked to patient outcomes to develop new AI-augmented digital pathology tools for MASLD/MASH. Using training and validation sets derived from the SteatoSITE cohort, new risk prediction indices derived from SHG/TPE imaging features were shown to predict all-cause mortality, decompensation events, and HCC, outperforming both NASH-CRN and qFibrosis ordinal staging [70].

Additionally, analysis of the SteatoSITE bulk RNA-seq data has enabled the discovery of molecular features linked to outcomes. In Kendall *et al*. [13], a 15-gene transcriptional risk score (TRS) was associated with a higher risk of developing decompensation events in advanced MASLD. Moreover, six of the 15 genes are predicted by bioinformatics to translate into secretome markers. The TRS was also used to investigate transcriptional regulatory networks in MASLD. Three regulons (gene networks controlled by AE binding protein 1 (AEBP1), thyroid hormone receptor beta (THRB), and basonuclin zinc finger protein 2 (BNC2)) exhibited significantly higher counts of TRS genes than anticipated by chance. This suggests that these three networks might play a crucial role in the progression of MASLD. Of particular interest, given recent encouraging data on the THRB agonist resmetirom [71], THRB regulon activity not only decreased with advancing fibrosis stage but also predicted future hepatic decompensation (beyond standard fibrosis scoring).

SteatoSITE was also used to perform deconvolution of the bulk RNA-seq using a published single-cell RNA-seq (scRNA-seq) reference dataset from healthy and cirrhotic patients [72], to estimate cell proportions in MASLD and correlate specific cell subpopulations with clinical outcomes. Interestingly, hepatic scar-associated macrophages (SAMacs) strongly correlated with fibrosis severity and were predictive for all-cause mortality and hepatic decompensation events. Conversely, more homeostatic liver resident cell types such as liver sinusoidal endothelial cells and vascular smooth muscle cells were protective against future mortality or hepatic decompensation.

Derived from a Scottish population with a high prevalence of MASLD and liver-related deaths [73], SteatoSITE is outcome-rich, but also has some specific limitations including inherent spectrum bias and a lack of ethnic diversity. Therefore, compared to other cohorts, SteatoSITE may be less suitable for modeling the population-level natural history of MASLD, and caution is advised about the generalizability of findings to other geographical areas and ethnic populations. Nevertheless, SteatoSITE is currently a unique resource for broad research efforts in MASLD including patient stratification, digital pathology methods, biomarker [74] and drug target discovery.

## 5 Technical challenges of using big data in MASLD research and practice

The main technical challenges can be categorized into two domains: those arising from using EHRs and those related specifically to AI. Prominent EHR challenges include interoperability and usability. Globally, EHR systems have different clinical terminologies and technical specifications [75], which can create barriers when exchanging and using the data, as both aspects need to be addressed to achieve true interoperability. Additional factors hampering the use of EHRs for research purposes include human error (e.g., incorrect data entry, typographical errors, sample mislabelling), difficulties with data standardization, errors arising from different delimiters or encoding in input files, issues related to data formatting, and instances of data duplication, missing data or incompleteness.

Despite the promise of AI/ML approaches in many aspects of MASLD research and clinical practice, certain technical challenges and limitations should be acknowledged. ML algorithms must undergo training to effectively identify patterns in the data. This process is hindered by the notoriously large dimensionality of features in medical datasets, referred to as the “curse of dimensionality”, often resulting in suboptimal algorithm performance in independent studies and failure to generalize to clinical scenarios. Additionally, it is easy to ignore that all input data are generated within a non-stationary environment with shifting patient populations that “drift” away from original training data. This phenomenon adversely affects algorithm performance and should be monitored and mitigated during live deployment. Furthermore, clinicians and pathologists, with differing expertise, contribute to the input data, which may therefore exhibit discrepancies in features/data for the model. Variability in obtaining input data, influenced by factors such as tissue quality, experimental locations and equipment, can contaminate feature selection and ground truthing and adversely impact model performance. AI systems, acting as black boxes (with internal workings that are invisible to the researchers/users), can perpetuate biases that are challenging to detect, such as hidden stratifications [76]. Transparency is therefore crucial in publishing AI models for reliability, reproducibility, and diagnostic use. Additionally, although somewhat theoretical at present, AI algorithms are susceptible to the risk of adversarial attack, which describes an otherwise effective model that can be manipulated by the provision of inputs explicitly designed to fool it and to purposefully generate an incorrect prediction [77]. Finally, standardization and regulatory approval would be essential for future clinical utilization of these diverse algorithms and models in disease diagnosis and assessment.

## 6 Future directions

The incorporation of AI/ML into MASLD research is swiftly advancing. By leveraging appropriate tools and methodologies, such as dimensionality reduction [78] and feature selection, data scientists can extract valuable insights from the growing complexity of accessible datasets. The assessment of liver histology using AI-augmented digital pathology tools is being assimilated into MASLD interventional trials, where digital analyses might provide better reproducibility and greater insights into drug efficacy and mechanism of action than standard scoring methods [79]. Moreover, the integration of AI-digital pathology with spatially resolved ‘omics data and clinical outcomes could drive the development of new histopathological-based metrics and refined categorizations for the stratification and prognostication of MASLD.

Finally, in the longer-term, AI might be applied in various ways to enhance clinical trials in MASLD. For example, AI algorithms could analyze EHRs to pinpoint eligible patients for clinical trials, improving patient recruitment efficiency, or be used to predict patient responses to treatment, helping in the selection of appropriate candidates for specific interventions. In addition, AI algorithms could continuously monitor patient data in real-time for early detection of adverse events, enhancing participant safety during the trial.

## Funding

The creation and initial analysis of SteatoSITE was funded by Innovate UK ((Precision medicine: impacting through innovative technology (Reference: TS/R017581/1; J.A.F. and T.J.K.)), Innovate UK Eureka (Reference: 105976; J.A.F., T.J.K. and I.D.), Guts UK Development Grant (Reference: DGO2019_16; J.A.F. and T.J.K.), industrial Collaborative Awards in Science and Engineering (iCASE) PhD studentship funded by the Medical Research Council Precision Medicine Doctoral Training Programme (Reference: MR/R01566X/1; M.J.R.) and Galecto Biotech (M.J.-R.).

## Declaration of interests

T.J.K. serves as a consultant for or has received speakers’ fees from Resolution Therapeutics, Clinnovate Health, Perspectum, Servier Laboratories, Kynos Therapeutics, and Incyte Corporation. J.A.F. serves as a consultant or advisory board member for Resolution Therapeutics, Kynos Therapeutics, Sosei Heptares, Ipsen, Redx Pharma, River 2 Renal Corp., Stimuliver, Galecto Biotech, Global Clinical Trial Partners and Guidepoint and has received research grant funding from Intercept Pharmaceuticals and Genentech. I.D. is a shareholder in Bering Limited.
